# Age estimation using methylation-sensitive high-resolution melting (MS-HRM) in both healthy felines and those with chronic kidney disease

**DOI:** 10.1038/s41598-021-99424-4

**Published:** 2021-10-07

**Authors:** Huiyuan Qi, Kodzue Kinoshita, Takashi Mori, Kaori Matsumoto, Yukiko Matsui, Miho Inoue-Murayama

**Affiliations:** 1 Wildlife Research Center, Kyoto University, Kyoto, 606-8203 Japan; 2 Kyoto Medical Center, Daktari Animal Hospital, Kyoto, 615-8234 Japan; 3 Tama Zoological Park, Tokyo, 191-0042 Japan; 4 Present Address: Miyazaki Prefectural Miyakonojo Livestock Hygiene Service Center, Miyazaki, 889-4505 Japan

**Keywords:** Ecology, Zoology, Biomarkers, Diseases, Epigenetics, DNA methylation

## Abstract

Age is an important ecological tool in wildlife conservation. However, it is difficult to estimate in most animals, including felines—most of whom are endangered. Here, we developed the first DNA methylation-based age-estimation technique—as an alternative to current age-estimation methods—for two feline species that share a relatively long genetic distance with each other: domestic cat (*Felis catus*; 79 blood samples) and an endangered *Panthera*, the snow leopard (*Panthera uncia*; 11 blood samples). We measured the methylation rates of two gene regions using methylation-sensitive high-resolution melting (MS-HRM). Domestic cat age was estimated with a mean absolute deviation (MAD) of 3.83 years. Health conditions influenced accuracy of the model. Specifically, the models built on cats with chronic kidney disease (CKD) had lower accuracy than those built on healthy cats. The snow leopard-specific model (i.e. the model that resets the model settings for snow leopards) had a better accuracy (MAD = 2.10 years) than that obtained on using the domestic cat model directly. This implies that our markers could be utilised across species, although changing the model settings when targeting different species could lead to better estimation accuracy. The snow leopard-specific model also successfully distinguished between sexually immature and mature individuals.

## Introduction

Age is an important ecological tool for wildlife conservation. As it is related to reproduction^1,2^ and mortality rates^3^, knowing the accurate age of an animal is helpful in estimating the structure of populations^4^ and, consequently, in the prediction of the present and future extinction risk for a wildlife population. Age estimation of wild-born individuals placed in captivity for their protection is beneficial for better health management and a more efficient reproduction schedule. However, most of the prevailing age-estimation methods have some deficiencies. For instance, estimating age through individual tracking, e.g. direct observation of primates^5^ and mark-recapture of bats^6^, is time consuming and difficult to apply to species that are difficult to observe and recapture. Observation of appearance change is also only possible for a limited number of species that exhibit easily observable and significant age-related changes, such as the accumulation of scars in cetaceans^7^. Methods based on observing the age-related development and eruption of teeth and bones can only be executed either on dead bodies or through capture and long-term restraint of living individuals for measurement or for taking dental photographs^8–10^.

Recently, an increasing number of studies have focused on molecular aging markers, which can be used to determine an individual’s age by sampling and analysing only a small amount of material. DNA methylation is one representative of molecular aging markers^3,11,12^. Changes in aging-associated DNA-methylation levels occur in CpG islands—CpG dinucleotides occur in clusters that are often present in gene promoter sites^13^. This change is a factor that determines the level and integrity of gene expression and results in loss of body function^14^. Previous studies have established DNA methylation as one of the most accurate age markers^3,11,12^. However, these studies are mainly limited to humans^12,15,16^ and mice^17,18^, with only a few studies on other species, such as dogs (*Canis familiaris*)^19,20^, wolves (*Canis lupus*)^19,20^, chimpanzees (*Pan troglodytes*)^21^, humpback whales (*Megaptera novaeangliae*)^22^, and Bechstein’s bats (*Myotis bechsteinii*)^23^.

The domestic cat (*Felis catus*) is both an important companion animal and a model animal for other felines; therefore, it could be said that the domestic cat is an ideal choice as the first DNA methylation-based age-estimation target in felines. Knowing a cat’s age helps individuals who adopt abandoned cats to take steps to prevent age-related diseases and improve cat welfare. Although estimating age by measuring the development and eruption of teeth is a widely used method in domestic cats, dental checks^24^ require a longer duration of restraint for obtaining dental photographs, which in turn places the individual under prolonged stress. It would be more convenient to determine the age of blood DNA, which can be obtained during regular health checks wherein blood sampling is required. In this way, with only a short duration of restraint, it may be possible to obtain age-related information together with the results of other biochemical and blood parameters. The model and markers designed for domestic cats are expected to be useful for other wild felines, as related species share similar age-related DNA methylation changes as implied by previous studies on humans and chimpanzees^12^, and on dogs and wolves^19^. To verify this hypothesis, we compared the sequences of age-estimation markers used in our models among all felines whose genome data were published. If the sequences were highly conserved, it was assumed that the markers would also have high applicability to other felines. To further verify this by experiment, we used the blood samples of the snow leopard (*Panthera uncia*) available to our team^25,26^, an endangered large cat species that shares long genetic distance with the domestic cat^27^, to test the markers and models developed with domestic cat samples. Although it is difficult to collect blood samples from the wild, it is still possible once an individual is captured. A more accurate age can be estimated by applying the methylation-based age-estimation method together with traditional dental morphology-based methods.

In this study, we included not only healthy individuals but also those with chronic kidney disease (CKD), one of the most common diseases in elderly felines^28,29^, to determine the influence of CKD on estimation accuracy. To our knowledge, our study presents the first age-estimation model applied to non-human species, including diseased individuals, by which we could devise specific ways to apply our model in the future. Specifically, if the estimation accuracy was influenced by CKD, it would imply that the markers should be used with a combination of disease biomarkers or pre-knowledge of the health condition of target individuals.

In this study, we developed a DNA methylation-based age-estimation technique for felines as a promising alternative to current age-estimation methods. We analysed the methylation rate of two epigenetic aging marker candidates (Table 1): (1) *ELOVL2*^30–32^ (elongation of very long chain fatty acid protein) and (2) *RALYL*^20^ (RALY RNA binding protein-like) via methylation-sensitive high-resolution melting (MS-HRM)^30,31,33–35^.

**Table 1:** Primer information and PCR conditions.

Gene	Primers	Length	n(CpGs)	PCR conditions	NCBI sequence ID: position	References
*ELOVL2*	F: TgTYgTYgYggYgTTTTTtgT R: ccaAaAAcRAAcRAcRAAtcc	117	13	95 ℃ (5 min), [95 ℃ (10 s); 55 ℃ (30 s); 72 ℃ (12 s)]*42 cycles	NC_018727.3: 17,965,149 to 17,965,265 (reverse complement)	^32^
*RALYL*	F: gCgatggttTtgtagaTaagg R: cgttttttccataaaaccaAttA	109	9	95 ℃ (5 min), [95 ℃ (10 s); 50 ℃ (30 s); 72 ℃ (10 s)]*45 cycles	NC_018740.3: 34,400,393 to 34,400,501	^20^

Capital letter: bisulfite-converted letter (F: c → T, R: g → A). Mixed base: Y = C + T, R = G + A.

## Results

### Standard curves

**Figure 1:**
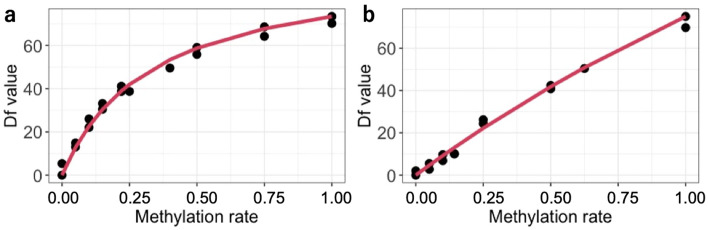
The standard curve of (**a**) *ELOVL2* (elongation of very long chain fatty acid protein) and (**b**) *RALYL* (RALY RNA binding protein-like) for domestic cat samples. Df value is the raw fluorescence data from methylation-sensitive high-resolution melting (MS-HRM). Data on snow leopard samples is shown in Supplementary Fig. S1.

In MS-HRM analysis, a standard curve was prepared for each experiment to calculate the methylation status of the samples, following Eq. (1) in the “Calculation of methylation rates” of the “Materials and methods” section. The standard curves of *ELOVL2* and *RALYL* in domestic cats are shown in Fig. 1. The standard curves of the snow leopards are shown in Supplementary Fig. S1. The estimated value of “a” in Eq. (1) for each species and each gene region are summarised in Supplementary Table S1.

### Correlation between methylation rates and age

**Figure 2:**
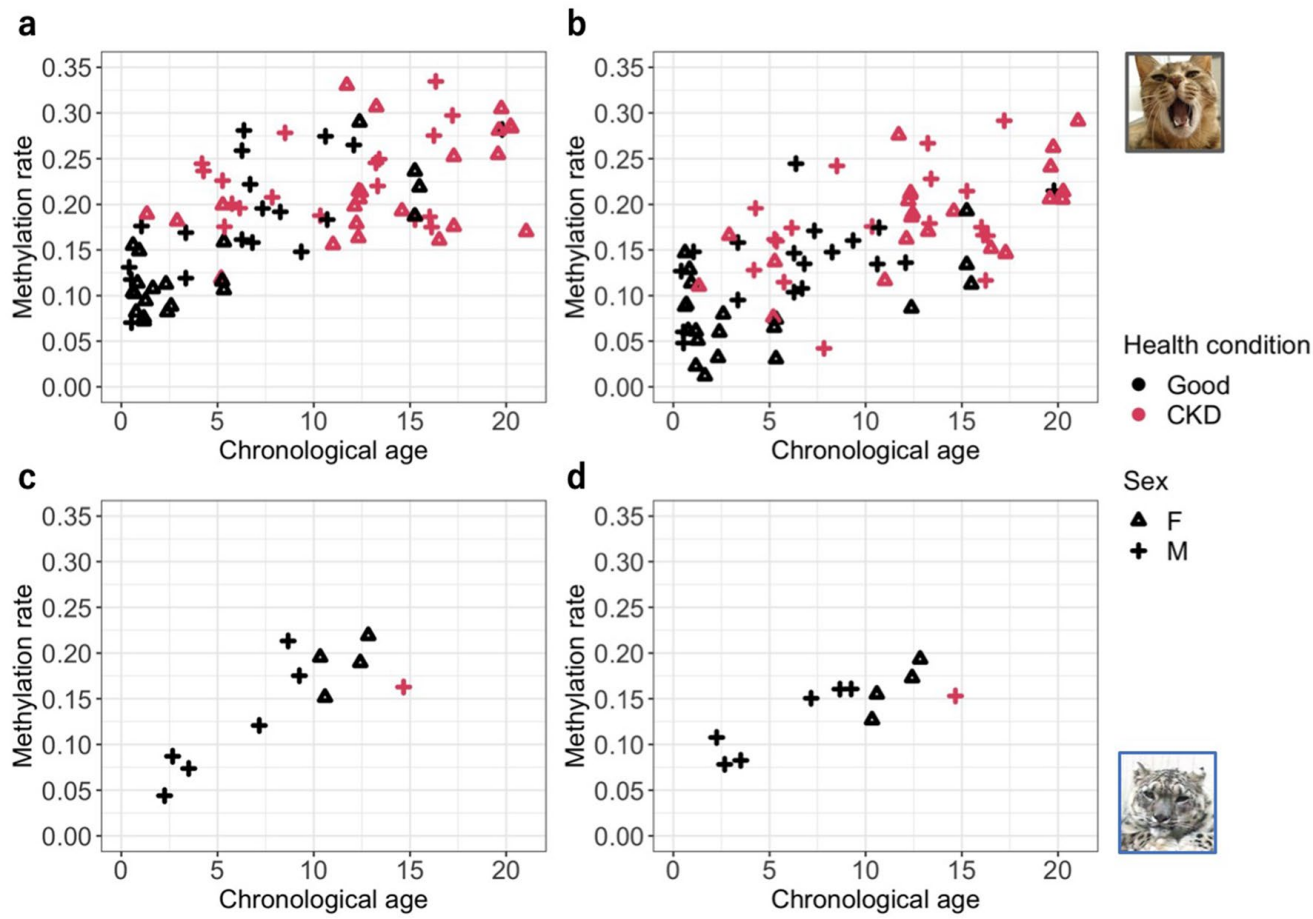
Correlation between methylation rate and chronological age for each gene region of each species: (**a**) domestic cat & *ELOVL2* (elongation of very long chain fatty acid protein), (**b**) domestic cat & *RALYL* (RALY RNA binding protein-like), (**c**) snow leopard & *ELOVL2*, and (**d**) snow leopard & *RALYL*. Domestic cat photo © H. Q., snow leopard photo © K. K.

The methylation rates of *ELOVL2* and *RALYL* in domestic cats were significantly correlated with age (*ELOVL2*: cor = 0.68, *p* < 0.001; *RALYL*: cor = 0.67, *p* < 0.001) (Fig. 2a, 2b). The results for the snow leopard were similar (*ELOVL2*: cor = 0.84, *p* < 0.01; *RALYL*: cor = 0.83, *p* < 0.01) (Fig. 2c, 2d).

### Age-estimation model

#### Model that used all domestic cat samples

**Figure 3:**
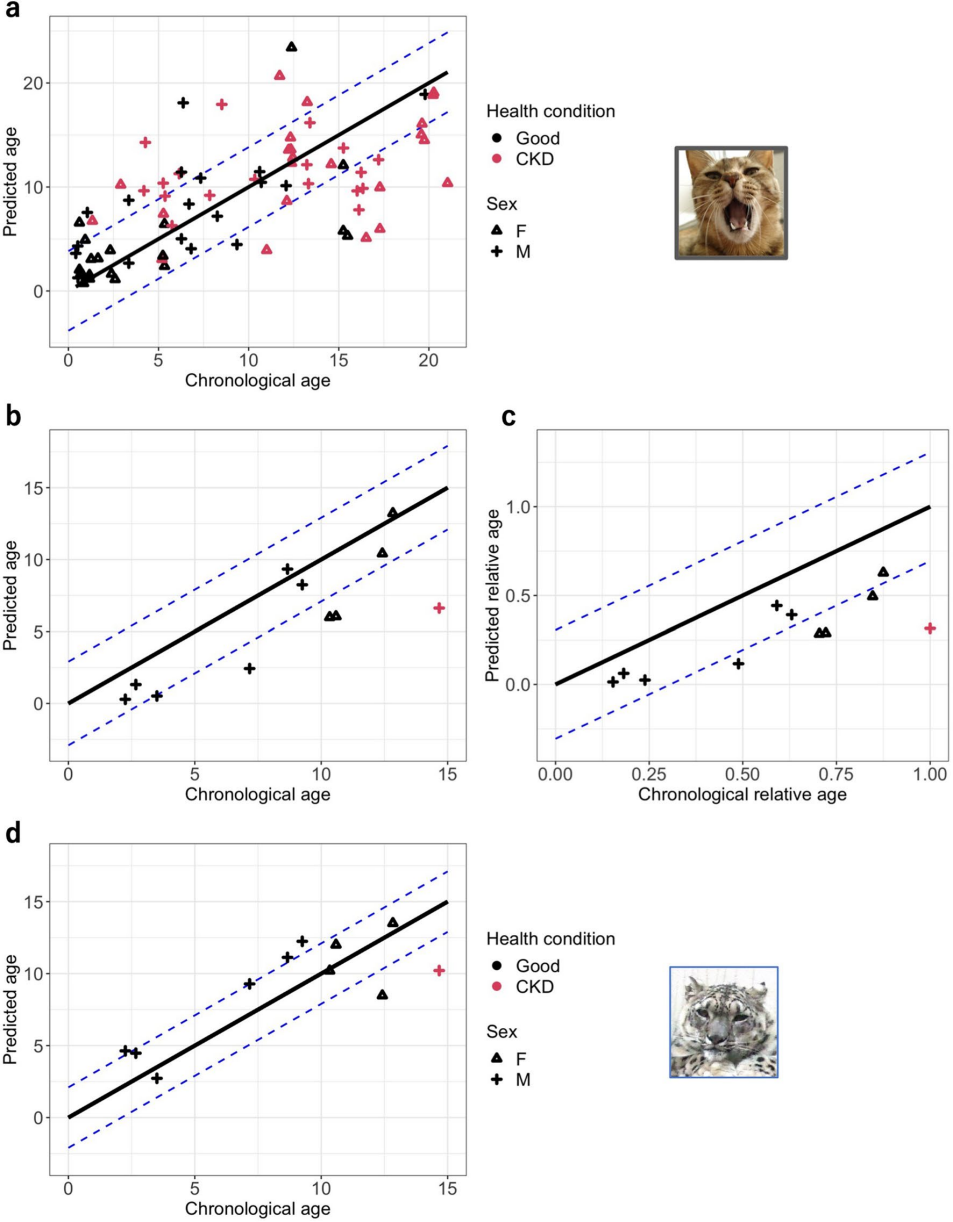
Correlation between predicted age and chronological age: (**a**) domestic cat, (**b**) snow leopard using the cat model directly. (**c**) Correlation between the predicted relative age and chronological relative age of snow leopards on using cat model directly. (**d**) Correlation between the predicted age and chronological age of snow leopards in snow leopard-specific model. The black line represents the y = x diagonal line. The region between blue dash lines of each plot was the mean absolute deviation (MAD) range: (**a**) MAD = 3.83 years, (**b**) MAD = 2.91 years, (**c**) MAD = 0.306 relative age, and (**d**) MAD = 2.10 years.

As the methylation rates of the two gene regions were correlated with age, both were used as explanatory variables in the age-estimation model. After leave-one-out validation (LOOCV) (Fig. 3a), the mean absolute deviation (MAD) was 3.83 years. To evaluate the source of the deviation of the model, we used linear regression to determine the factors that contributed to the estimated age difference (i.e. the difference between the predicted age and chronological age). As a result, the best regression model (R^2^ = 0.41) included age, sex, and health conditions as explanatory variables. Male individuals and those with CKD had larger estimated age differences, whereas older individuals had smaller estimated age differences (Table 2). Although other unknown factors might also contribute to the estimated age difference based on the low R^2^, the factors found in this model are still important.

**Table 2:** Coefficient value with *p* value in the linear regression model of estimated age difference which was got from the model using all domestic cat samples.

	Estimate	*P-value*
(Intercept)	2.00	< 0.01**
Age	− 0.43	< 0.001***
Sex (M)	1.88	< 0.01**
Health condition (CKD)	2.03	< 0.05*

*CKD* chronic kidney disease.

#### Models for different sex and health condition combinations

As both sex and health conditions contributed to the estimated age differences obtained from the model that used all samples, the age-estimation accuracy was expected to improve when separate estimation models were built for different sex and health condition combinations (healthy females: 20 samples; CKD females: 23 samples; healthy males: 18 samples; CKD males: 18 samples). The estimation accuracy was improved in the models of females, especially in the model of healthy females with MAD after LOOCV at 2.93 years (Table 3). For the male models, MAD improved before LOOCV (Table 3); however, after LOOCV, the accuracy degraded significantly (Table 3).

**Table 3:** Mean absolute deviation (MAD) results for each domestic cat model with different sex and health condition combinations.

The target of models	MAD (year)	MAD (year) (after LOOCV)
Healthy female	1.03	2.93
CKD female	0.94	3.76
Healthy male	2.20	4.63
CKD male	2.20	5.08

*CKD* chronic kidney disease, *LOOCV* leave-one-out validation.

#### Snow leopard models

First, snow leopard samples were used as a test set to validate whether the model developed on domestic cats (Fig. 3a) could be directly used for snow leopards. MAD was 2.91 years for snow leopards. Although the accuracy was similar to that of domestic cats, the age of most samples was underestimated (Fig. 3b). On examining the model of relative age, which is the ratio of chronological age to the age of the oldest individual of the respective species in our study, the underestimation was worse (Fig. 3c, MAD = 0.306 relative age). Next, we reset the model parameters (Supplementary Table S2) for snow leopards, and the accuracy was significantly improved (Fig. 3d; after LOOCV, MAD = 2.10 years). On rebuilding the model using only healthy samples (i.e. excluding one CKD sample), the estimation accuracy was at the same level as that in the snow leopard-specific model using all samples (after LOOCV, MAD = 2.02 years).

#### The potential use of markers in other felines

**Figure 4:**
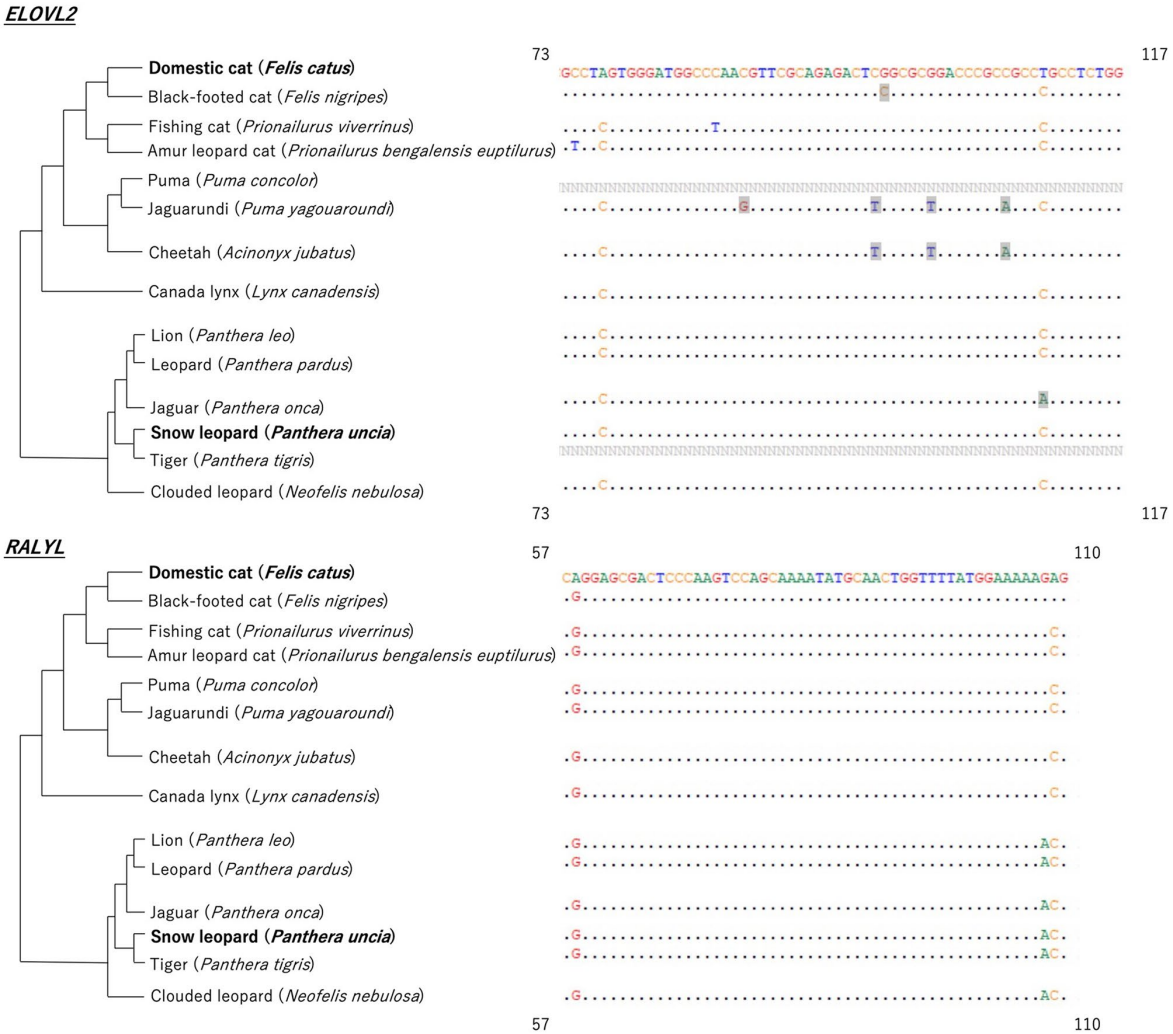
The alignment of feline *ELOVL2* (elongation of very long chain fatty acid protein) and *RALYL* (RALY RNA binding protein-like) marker regions with feline phylogenetic trees. Only the parts of sequence where differences between felines appear are shown, with starting and ending positions shown in the four corners of the alignments. Bases highlighted in grey were located in the domestic cat’s and the snow leopard’s CpG sites, which were not conserved in the relevant species.

Data for the puma and tiger were absent from the database of the *ELOVL2* region (Fig. 4). The majority of the sequences among the felines were the same, although there was a decrease in some CpG sites (i.e. bases highlighted in grey) compared to that in either the domestic cat or the snow leopard. For the *RALYL* region, although there were some differences in some bases, the CpG sites were identical to those of either the domestic cat or the snow leopard.

## Discussion

This is the first epigenetic age-estimation study in felines and includes both healthy and diseased individuals. The age-estimation model of domestic cats predicted age from blood samples with an MAD value of 3.83 years (i.e. 19.3% of the oldest age) (Fig. 3a). The estimation accuracy was further improved to 2.93 years when only healthy female samples were targeted (i.e. 18.9% of the oldest age). The estimation accuracy of our study was lower than that of previous studies in other species using similar targeted bisulfite sequencing^22,23^ (approximately 10% of the oldest age). This is not due to the difference in the accuracy of methods; for MS-HRM was reported to share an accuracy similar to that of pyrosequencing^36,37^, which is the major targeted bisulfite sequencing method used in previous studies^22,23^. The relatively low estimation accuracy is possibly caused by the limited number of age-estimation markers used in our study. It is hoped that the accuracy may be further improved by increasing the number of markers. MS-HRM is a real-time PCR-based technique, which is easier, quicker (almost 2 h for each run), and more cost-effective (costs $7 per sample for age estimation based on two markers) than pyrosequencing (needs 3–4.5 h for each run, and costs $14 per sample for age estimation based on two markers)^23,38^. The disadvantage of MS-HRM is that only the average methylation rate of target regions, but not that of individual CpG sites, could be known, whereas that of individual CpG sites is not that necessary to know in clinical measurement. Overall, for domestic cats, this study provided a new possibility to estimate the age of an adopted cat from blood that could be obtained rapidly during regular health checks, as an alternative to dental check-ups that require a longer time of restraint^24^ and are limited to some age stages^39^.

As diseases have been reported to affect DNA methylation levels in some regions^40,41^, clarifying whether diseases affect selected genetic markers could contribute to a more applicable and accurate age-estimation model. This implies that, if the markers were found to be influenced, knowing the health condition of the targeted individuals and estimating the age of healthy and diseased individuals separately would be necessary. However, it is difficult to find practical studies on age estimation to study the influence of health conditions on model accuracy, in which the target species are neither humans nor mice. Furthermore, no previous research has investigated whether CKD influences the methylation levels of *ELOVL2* and *RALYL* in blood. In our study, we found that the estimation accuracy was affected by health conditions, and the estimation accuracy of the healthy individuals’ model was higher than that of the CKD model. CKD had different levels of influence on health at different age stages, which could consequently decrease the estimation accuracy. CKD is a common disease in older felines and might affect over 30%–40% of cats over 10 years of age^28,29^; therefore, it is reasonable to assume that older CKD individuals could still have normal health conditions and, thus, a normal physiological/predicted age, whereas it is the opposite in young individuals with CKD. Therefore, for CKD females, most of whom were older individuals (> 10 years) (Fig. 3a), CKD did not have a significant influence on the estimation accuracy. However, in the CKD male group with both young and old individuals in equal proportions (Fig. 3a), CKD had different influences at different age stages, which led to a low estimation accuracy. The higher ratio of young individuals in the CKD male group than in the CKD female group is also consistent with the report that male cats are more frequently diagnosed with CKD than females at a young age^42^. For future studies, other common feline diseases should also be considered to determine their influence on the age-estimation model.

For the results of snow leopards, the estimation accuracy was much improved after resetting model parameters for snow leopards, i.e. MAD changed from 2.91 years (Fig. 3b) to 2.10 years (Fig. 3d), which implies that although the markers were common in the two related species, the model could be species-specific. Therefore, for further application to other feline species, it might be necessary to reoptimize the model settings for each species. We also successfully distinguished young individuals (i.e. those around 2–3 years old) and other elderly individuals (Fig. 3d). Based on the life cycle information from captive^1^ and wild^43^ snow leopards, it is reported that these animals become sexually mature around 2–3 years of age. Thus, it could be said that our model can help to distinguish between individuals at the reproductive stage and those who are not. This would, in turn, contribute to both wild- and captive-population management. The greatest deficiency of our snow leopard model was the small sample size (11 samples). In future studies, more snow leopard samples should be used to evaluate our snow leopard model and analyse the influence of sex and health conditions on the model, which could not be analysed in our study due to the small sample size.

Although we only studied domestic cats and snow leopards, we found that the two gene regions we used were also highly conserved in other felines, which implies high applicability of our markers in all felines. The slight sequence difference between species implied a slightly different methylation background. This also implied that, although the markers could be widely applied, the age-estimation model could be species-specific or specific to those sharing exactly the same sequence. One caution is that for the black-footed cat, the PUMA lineage (i.e. puma, jaguarundi, cheetah), and the jaguar, the number of CpG sites in *ELOVL2* was slightly less than that in the domestic cat and the snow leopard. The MS-HRM method could only determine the average methylation of the whole target region, therefore we could not determine which CpG sites were important and whether they were conserved in these species or not. Thus, future studies should verify whether the *ELOVL2* marker is usable in black-footed cats, the PUMA lineage, and the jaguar before applying it to these species.

In addition, when this method is applied to wild animals, it is important to determine whether the environment and difference in food resources between captive and wild individuals could influence the model function. Some previous studies have implied that DNA methylation of some gene regions is influenced by these external factors^44,45^. For future utilisation in wild individuals, more robust markers that are not easily influenced by the environment or food resources should be selected.

## Materials and methods

### Ethics statement

All methods were carried out in accordance with relevant guidelines and regulations. The study was carried out in compliance with the ARRIVE guidelines. All experimental protocols were approved by the ethical committee of Wildlife Research Center of Kyoto University and all sample collection and experiments were conducted with permission from the ethical committee with approval number WRC-2019-012A, WRC-2020-012A and WRC-2021-001A. All domestic cat samples were obtained in agreement with cat owners. All snow leopard samples were collected with the approval of each zoo. Data anonymization was done for all the samples.

### Sample collection and DNA extraction

Residual blood samples from health check-ups of 79 domestic cats were collected from the Daktari Animal Hospital and Anicom Specialty Medical Institute Inc. between July and September 2020. Information on age, cat breed, neuter status, sex, and health condition was recorded by the veterinarians. The cats were either healthy or had CKD. The diagnosis of CKD followed the IRIS CKD guidelines wherein serum creatinine and symmetric dimethylarginine (SDMA) in blood were both used as diagnostic markers^46^. All domestic cat samples were stored at − 80 °C for less than 1 month before DNA extraction. Most domestic cat samples were obtained from mixed breed individuals (n = 52). The remaining 27 individuals were pure breed cats (British Shorthair, 3; Chinchilla, 1; Chinchilla Persian; 1; exotic Shorthair, 4; Japanese cat, 2; Munchkin, 4; Norwegian Forest Cat, 6; Russian Blue, 2; Scottish Fold, 3; Somali, 1). As there were only a few samples for pure breed cats, breed was not considered an important factor that could have influenced the methylation rates. Of these, 25 individuals were unneutered, most of whom were younger than 2 years of age. The age distributions with different sex and health condition combinations are shown in Supplementary Table S3. The sex ratio (F:M) was 43:36, age ranged from 0.41 to 21.04 years, and the number of healthy individuals was 38. The other patients had CKD (n = 41).

For snow leopards, 11 samples from individuals between 2.25 and 14.67 years of age were used. Seven individuals were male and four were female. The age and sex information were summarised in Supplementary Table S4. According to the diagnosis of veterinarians, all individuals were in good health condition, except for the oldest individual who was diagnosed with CKD based on blood tests but did not exhibit significant symptoms. These samples were stored at –20 ℃ before DNA extraction. DNA from all the above samples of both species was extracted using the DNeasy Blood & Tissue Kit (QIAGEN GmbH, Hilden, Germany) and stored at –20 °C until use.

### Standard DNA

Standard DNA is needed in MS-HRM to calculate the methylation status of the samples. A 0% methylated standard DNA was obtained by performing whole genome amplification treatment on one DNA sample from each species using the REPLI-g Mini Kit (QIAGEN GmbH, Hilden, Germany). We obtained 100% methylated standard DNA by fully methylating the same DNA sample with CpG methyltransferase (*M.SssI*; New England Biolabs, Beverly, MA, USA). Standard DNA (0% and 100%) was purified using a High Pure PCR Product Purification Kit (Roche Molecular Systems, Pleasanton, CA, USA).

### Bisulfite conversion

DNA samples and purified standard DNA were bisulfite-converted using the EZ DNA Methylation-Gold Kit (Zymo Research, Irvine, CA, USA). The concentration of bisulfite-converted DNA was then measured with a Qubit 4 Fluorometer using a Qubit ssDNA Assay Kit (Thermo Fisher Scientific, San Jose, CA, USA) and finally adjusted to 5 ng/μL.

### Gene regions and primer design

The target genes were *ELOVL2* (elongation of very long chain fatty acids protein 2) and *RALYL* (RALY RNA binding protein-like). *ELOVL2* is a well-known biomarker of aging, which has been widely reported in humans^30–32^, chimpanzees^12^, and mice^47^. Although there are few studies on *RALYL*, it has been reported to be hypermethylated in cancer tissues^48,49^ and was used as a biomarker by Lowe et al. (2018)^20^ to estimate the age of domestic dogs, the carnivorous relatives of felids^20^. Homogeneous gene regions were blasted in the domestic cat genome (GCA_000181335.4) provided by NCBI^50^ via BLASTN^51^. PCR primers were designed using Methyl Primer Express v1.0 (Thermo Fisher Scientific, San Jose, CA, USA; Table 1). The same primers and conditions were used for both species.

### Methylation-sensitive high-resolution melting (MS-HRM)

In MS-HRM, bisulfite-converted DNA samples were PCR-amplified, followed by melting analysis. PCR amplification was carried out using a Roche LightCycler 480 Instrument II (Roche Molecular Systems, Branchburg, NJ, USA) equipped with the Gene Scanning Software 96 (version 1.5.1.62 SP2) in a 25 μL total volume containing 1 × EpiTect HRM PCR Master Mix (EpiTect HRM PCR Kit; QIAGEN GmbH, Hilden, Germany), 750 nM of each primer, and 2 μL of template DNA (5 ng/μL bisulfite-converted DNA). The PCR conditions are presented in Table 1. After PCR amplification, samples were cooled to 65 °C for 1 s and then heated to 95 °C at 0.02 °C/s to melt gradually. The continuous fluorescence data was gained at 25 acquisitions/s during the entire process. Standard curves with known methylation ratios (0%, 5%, 10%, 15%, 25%, 40%, 50%, 75%, and 100%) were included in each assay and were later used to calculate the methylation rate of each sample. The standard series was prepared by mixing 0% methylated standard DNA and 100% unmethylated standard DNA in appropriate ratios. All reactions were performed in duplicate.

After the experiment, the melting curves were normalised relative to the two temperature regions before and after the major fluorescence decrease. We set the pre-melt temperature region to 66–68 °C and the post-melt temperature region to 84.5–85 ℃ for *ELOVL2*. For *RALYL*, the pre-melt temperature region was 67–68 °C and the post-melt temperature region was 82–83 °C.

The difference curves (Supplementary Fig. S2) were then derived from the first derivative of the melt curves after setting the data of the 0% methylated standard sample as a baseline. The maximum absolute value of the relative signal difference from the difference curves were defined as “Df value” (Supplementary Fig. S2), for each sample.

### Calculation of methylation rates

The Df values of the standard series were plotted for each species, gene region, and plate. The standard curve follows a non-linear regression model designed by Hamano et al. (2016)^30^ as follows:

1 $$\frac{\mathrm{a}*\mathrm{M}}{100-\mathrm{M}}= \frac{\mathrm{Df}}{{\mathrm{Df}}_{\mathrm{max}}-\mathrm{Df}}$$

where M is methylation rate, Df_max_ is the Df value of 100% methylated standard sample and “a” is a coefficient. The conduction of the regression model and the calculation of the estimated value of "a" were carried out using the nls command in R 4.0.3. The methylation rates of the samples were calculated later by substituting the Df value into Eq. (1).

### Age-estimation model and model validation

Before building the model, we calculated Pearson's product-moment correlation coefficients with *p* values between age and the methylation rate of each target region. To build the age-estimation models, we used support vector regression (SVR), which is implied by Xu et al.^52^, to be a robust choice that has high estimation accuracy with a low-level overfit. The R package “e1071”^53^ was used to build the models. The parameters of the SVR models were optimised using the “tune” command with optimizable parameters “cost” and “epsilon” and fixed settings “type = eps-regression, kernel = radial, gamma = 0.5”. The optimised parameters are summarised in Supplementary Table S2. LOOCV was used to validate the overfitting of the optimised models. To evaluate the source of the deviation in the model that used all domestic cat samples, a linear regression model with the age-estimation difference (predicted age − chronological age) as the response variable was built using the “lm” command. The model selection was conducted with the “MuMIn” package.

### Sequences of the target regions in other felines

We also compared the similarity of our target regions among the felines with published genome data. We used the sequences of domestic cats as queries and performed a BLASTN search in the feline genome database (i.e. NCBI^50^ or DNA Zoo Consortium^54^). The sequence data where differences between species appeared were summarised with phylogenetic trees of felines^27^ (Fig. 4).

## Supplementary information

Supplementary Information 1.

## Data Availability

The raw data and R script of this study can be accessed from the DOI (10.5061/dryad.66t1g1k2t).

## References

[1] Blomqvist, L. & Sten, I. *Reproductive Biology of the Snow Leopard*. Panthera Books, London (1982).

[2] Kirkwood TB, Austad SN (2000). Why do we age?. Nature.

[3] Zhao M, Klaassen CAJ, Lisovski S, Klaassen M (2019). The adequacy of aging techniques in vertebrates for rapid estimation of population mortality rates from age distributions. Ecol. Evol..

[4] Oli MK, Dobson FS (2003). The relative importance of life-history variables to population growth rate in mammals: Cole’s prediction revisited. Am. Nat..

[5] Mori A (1979). Analysis of population changes by measurement of body weight in the Koshima troop of Japanese monkeys. Primates.

[6] WILkINSON GS, Brunet-Rossinni AK (2009). Methods for age estimation and the study of senescence in bats. Ecological and behavioral methods for the study of bats.

[7] Hartman KL, Wittich A, Cai JJ, van der Meulen FH, Azevedo JMN (2016). Estimating the age of Risso’s dolphins (*Grampus griseus*) based on skin appearance. J. Mammal..

[8] Chevallier C, Gauthier G, Berteaux D (2017). Age estimation of live arctic foxes Vulpes lagopus based on teeth condition. Wildl. Biol..

[9] White PA (2016). Age estimation of African lions Panthera leo by ratio of tooth areas. PloS One.

[10] Siegal-Willott J, Isaza R, Johnson R, Blaik M (2008). Distal limb radiography, ossification, and growth plate closure in the juvenile Asian elephant (*Elephas maximus*). J. Zoo Wildl. Med..

[11] Paoli-Iseppi D (2017). Measuring animal age with DNA methylation: From humans to wild animals. Front. Genet..

[12] Horvath S (2013). DNA methylation age of human tissues and cell types. Genome Biol..

[13] Schübeler D (2015). Function and information content of DNA methylation. Nature.

[14] Field AE (2018). DNA methylation clocks in aging: Categories, causes, and consequences. Mol. Cell.

[15] Weidner CI (2014). Aging of blood can be tracked by DNA methylation changes at just three CpG sites. Genome Biol..

[16] Bocklandt, S. *et al.* Epigenetic predictor of age. *PloS One* **6**, e14821 (2011). 10.1371/journal.pone.0014821 PMC3120753 21731603

[17] Petkovich DA (2017). Using DNA methylation profiling to evaluate biological age and longevity interventions. Cell Metab..

[18] Stubbs TM (2017). Multi-tissue DNA methylation age predictor in mouse. Genome Biol..

[19] Thompson MJ, vonHoldt B, Horvath S, Pellegrini M (2017). An epigenetic aging clock for dogs and wolves. Aging (Albany NY).

[20] Lowe R (2018). Ageing-associated DNA methylation dynamics are a molecular readout of lifespan variation among mammalian species. Genome Biol..

[21] Ito H, Udono T, Hirata S, Inoue-Murayama M (2018). Estimation of chimpanzee age based on DNA methylation. Sci. Rep..

[22] Polanowski AM, Robbins J, Chandler D, Jarman SN (2014). Epigenetic estimation of age in humpback whales. Mol. Ecol. Resour..

[23] Wright PG (2018). Application of a novel molecular method to age free-living wild Bechstein’s bats. Mol. Ecol. Resour..

[24] Park K (2014). Determining the age of cats by pulp cavity/tooth width ratio using dental radiography. J. Vet. Sci..

[25] Yoshimura, H. *et al.* The relationship between plant-eating and hair evacuation in snow leopards (*Panthera uncia*). *PLOS ONE* **15**, e0236635 (2020). 10.1371/journal.pone.0236635 PMC7394552 32736376

[26] Kinoshita, K. *et al.* Long-term monitoring of fecal steroid hormones in female snow leopards (*Panthera uncia*) during pregnancy or pseudopregnancy. *PLOS ONE* **6**, e19314 (2011). 10.1371/journal.pone.0019314 PMC3085513 21559303

[27] Li G, Davis BW, Eizirik E, Murphy WJ (2016). Phylogenomic evidence for ancient hybridization in the genomes of living cats (Felidae). Genome Res..

[28] Marino CL, Lascelles BDX, Vaden SL, Gruen ME, Marks SL (2014). Prevalence and classification of chronic kidney disease in cats randomly selected from four age groups and in cats recruited for degenerative joint disease studies. J. Feline Med. Surg..

[29] Sparkes AH (2016). ISFM consensus guidelines on the diagnosis and management of feline chronic kidney disease. J. Feline Med. Surg..

[30] Hamano Y (2016). Forensic age prediction for dead or living samples by use of methylation-sensitive high resolution melting. Leg. Med..

[31] Hamano Y, Manabe S, Morimoto C, Fujimoto S, Tamaki K (2017). Forensic age prediction for saliva samples using methylation-sensitive high resolution melting: exploratory application for cigarette butts. Sci. Rep..

[32] Bekaert B, Kamalandua A, Zapico SC, Van de Voorde W, Decorte R (2015). Improved age determination of blood and teeth samples using a selected set of DNA methylation markers. Epigenetics.

[33] Hussmann, D. & Hansen, L. L. Methylation-sensitive high resolution melting (MS-HRM). In *DNA Methylation Protocols* (ed. Tost, J.) vol. 1708, pp. 551–571 (Springer New York, 2018). 10.1007/978-1-4939-7481-8_28 29224163

[34] Wojdacz, T. K. & Dobrovic, A. Methylation-sensitive high resolution melting (MS-HRM): A new approach for sensitive and high-throughput assessment of methylation. *Nucleic Acids Res.* **35**, e41 (2007). 10.1093/nar/gkm013 PMC1874596 17289753

[35] Mawlood SK, Dennany L, Watson N, Pickard BS (2016). The EpiTect methyl qPCR assay as novel age estimation method in forensic biology. Forens. Sci. Int..

[36] Migheli, F. *et al.* Comparison study of MS-HRM and pyrosequencing techniques for quantification of APC and CDKN2A gene methylation. *PLOS ONE* **8**, e52501 (2013). 10.1371/journal.pone.0052501 PMC3543439 23326336

[37] Xiao Z (2014). Validation of methylation-sensitive high-resolution melting (MS-HRM) for the detection of stool DNA methylation in colorectal neoplasms. Clin. Chim. Acta.

[38] Šestáková Š, Šálek C, Remešová H (2019). DNA methylation validation methods: A coherent review with practical comparison. Biol. Proc. Online.

[39] Fleming, P. A., Crawford, H. M., Auckland, C. & Calver, M. C. Nine ways to score nine lives—Identifying appropriate methods to age domestic cats (*Felis catus*). *J. Zool.*

[40] Smyth LJ, McKay GJ, Maxwell AP, McKnight AJ (2014). DNA hypermethylation and DNA hypomethylation is present at different loci in chronic kidney disease. Epigenetics.

[41] Chen, J. *et al.* Elevated Klotho promoter methylation is associated with severity of chronic kidney disease. *PloS One* **8**, e79856 (2013). 10.1371/journal.pone.0079856 PMC3818221 24224012

[42] White JD, Norris JM, Baral RM, Malik R (2006). Naturally-occurring chronic renal disease in Australian cats: A prospective study of 184 cases. Aust. Vet. J..

[43] Snow Leopard Trust. Snow leopard facts/life cycle. *Snow Leopard Trust* http://snowleopard.org/snow-leopard-facts/life-cycle/.

[44] Dhingra R, Nwanaji-Enwerem JC, Samet M, Ward-Caviness CK (2018). DNA methylation age—Environmental influences, health impacts, and its role in environmental epidemiology. Curr. Environ. Health Rep..

[45] Lea AJ, Altmann J, Alberts SC, Tung J (2016). Resource base influences genome-wide DNA methylation levels in wild baboons (*Papio cynocephalus*). Mol. Ecol..

[46] IRIS. IRIS Kidney—Guidelines—IRIS Staging of CKD. http://www.iris-kidney.com/guidelines/staging.html (2019).

[47] Spiers H (2016). Age-associated changes in DNA methylation across multiple tissues in an inbred mouse model. Mech. Ageing Dev..

[48] Vignettes, C.-B. *Proceedings from the 2015 Annual Meeting of the American College of Physicians*, Wisconsin Chapter. *WMJ* (2015).

[49] Zhang, X. *et al.* Genome-wide analysis of cell-free DNA methylation profiling with MeDIP-Seq identified potential biomarkers for colorectal cancer (2021). 10.1186/s12957-022-02487-4 PMC8783473 35065650

[50] MD, B., US, N. L. of M. & US, N. C. for B. I. National Center for Biotechnology Information (NCBI). https://www.ncbi.nlm.nih.gov/.

[51] Altschul SF, Gish W, Miller W, Myers EW, Lipman DJ (1990). Basic local alignment search tool. J. Mol. Biol..

[52] Xu C (2015). A novel strategy for forensic age prediction by DNA methylation and support vector regression model. Sci. Rep..

[53] Chang C-C, Lin C-J (2011). LIBSVM: a library for support vector machines. ACM Trans. Intell. Syst. Technol. (TIST).

[54] Dudchenko O (2017). De novo assembly of the *Aedes aegypti* genome using Hi-C yields chromosome-length scaffolds. Science.

