# Supplementary material for: Age estimation using methylation-sensitive high-resolution melting (MS-HRM) in both healthy felines and those with chronic kidney disease
Source: Sci Rep. 2021 Oct 7;11:19963. doi: 10.1038/s41598-021-99424-4 (PMC8497492; doi:10.1038/s41598-021-99424-4)
Supplement: Supplementary file 1 — Supplementary Information 1. [file 41598_2021_99424_MOESM1_ESM.pdf]

Age estimation using methylation-sensitive high-resolution melting (MS-HRM) in both healthy felines and those with chronic kidney disease

Huiyuan Qi[1], Kodzue Kinoshita[1], Takashi Mori[2], Kaori Matsumoto[2⁋], Yukiko Matsui[3], Miho Inoue-Murayama[1]*

[1]Wildlife Research Center, Kyoto University, Kyoto 606-8203, Japan

[2] Kyoto Medical Center, Daktari Animal Hospital, Kyoto 615-8234, Japan

[3]Tama Zoological Park, Tokyo 191-0042, Japan

⁋Current address: Miyazaki Prefectural Miyakonojo Livestock Hygiene Service Center, Miyazaki 889-4505, Japan

*Corresponding author:

Wildlife Research Center, Kyoto University, Kyoto 606-8203, Japan;

Phone: +81-75-771-4375;

Email: murayama.miho.5n@kyoto-u.ac.jp

Supplementary Table S1. Values of the coefficient "a" in equation (1) and R^2 (R-squared) of the standard curve for each species and gene region.

	**Domestic cat**	**Snow leopard**
ELOVL2	a = 3.40***<br>$R^2 = 0.99$	a =1.78***<br>$R^2 = 0.98$
RALYL	a = 0.91***<br>$R^2 = 0.98$	a =0.62***<br>$R^2 = 0.99$

*** $p < 0.001$

Supplementary Table S2. Optimised parameters of support vector regression (SVR) models.

	**Cost**	**Epsilon**
**Cat model (all samples)**	25.12	0.20
**Healthy female cat model**	63.10	0.20
**CKD female cat model**	25.12	0.10
**Healthy male cat model**	10.00	0.40
**CKD male cat model**	4.70	0.10
**Snow leopard-specific model (all samples)**	31.6	0.10
**Snow leopard-specific model (healthy samples)**	20.0	0.10

Supplementary Table S3. Age distribution of cat samples based on sex and health condition.

	Female			Male			Total
	Healthy	CKD	Total	Healthy	CKD	Total	
**Kitten**	0	0	**0**	3	0	**3**	**3**
**Junior**	12	1	**13**	1	0	**1**	**14**
**Prime**	4	3	**7**	5	6	**11**	**18**
**Mature**	0	0	**0**	5	3	**8**	**8**
**Senior**	1	9	**10**	3	3	**6**	**16**
**Geriatric**	3	4	**7**	0	6	**6**	**13**
**Long-living**	0	6	**6**	1	0	**1**	**7**
**Total**	20	23	**43**	18	18	**36**	**79**

Age stages follow a convenient life-stage classification adapted by AAFP-AAHA Feline Life Stage Guidelines 2010[1] with a newly defined long-living stage. Kitten refers to individuals under 6 months; junior to 6 months to 2 years old; prime to 3–6 years old; mature to 7–10 years old; senior to 11–14 years old; geriatric to 15–19 years old; long-lived refers to those over 19 years old.

1. Vogt, A. H. et al. AAFP–AAHA Feline Life Stage Guidelines Journal of the American Animal Hospital Association **46**, 16 (2010).

Supplementary Table S4. Age and sex information for the snow leopard samples.

Individual ID	Age	Sex
**1**	2.25	M
**2**	2.67	M
**3**	3.5	M
**4**	7.17	M
**5**	8.67	M
**6**	9.25	M
**7**	10.33	F
**8**	10.58	F
**9**	12.42	F
**10**	12.83	F
**11**	14.67	M

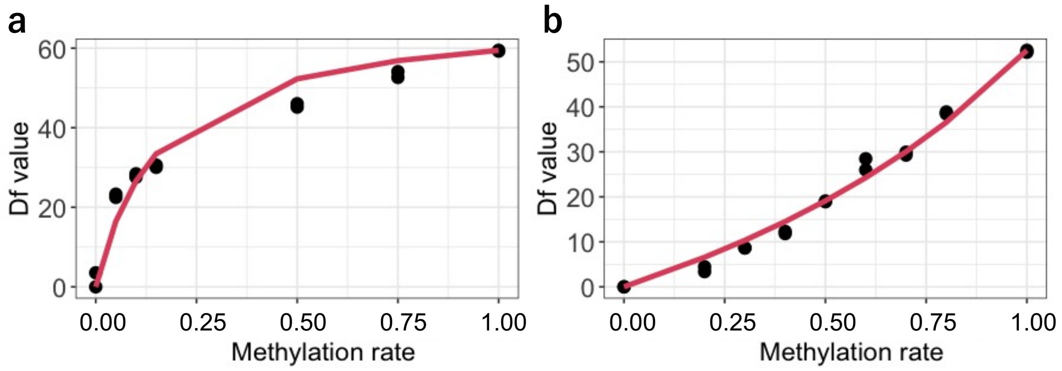

Supplementary Figure S1. The standard curves of snow leopards (*ELOVL2*: a, *RALYL*: b).

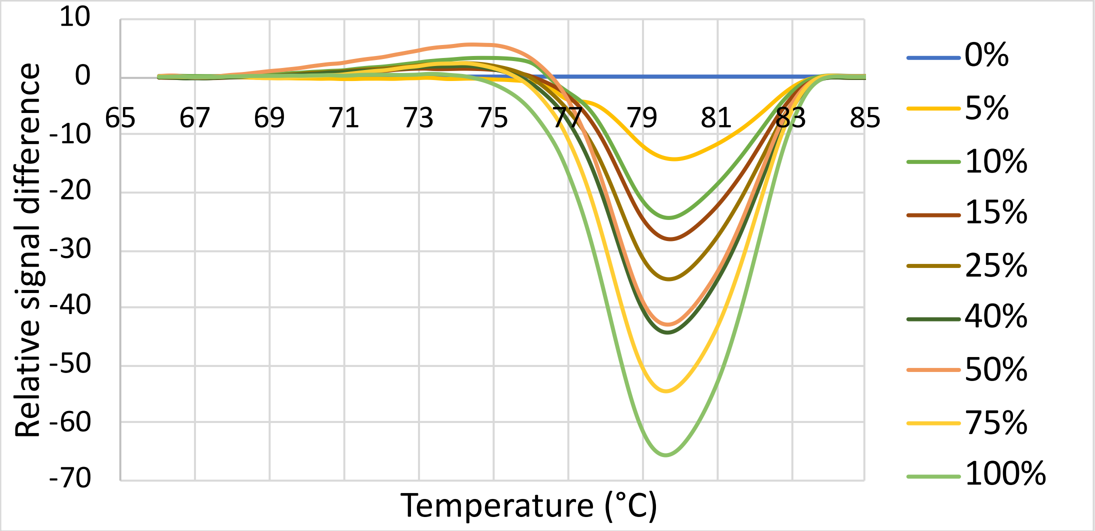

Supplementary Figure S2. The example of difference curves in MS-HRM analysis. Only the curves of standard samples are shown here as examples. Those of other samples had similar curve pattern. The data of 0% methylated standard sample was the baseline. The maximum absolute value of the relative signal difference from each difference curve were defined as “Df value” for each sample.
